# open-japanese-mesh: assigning MeSH UIDs to Japanese medical terms via open Japanese-English glossaries

**DOI:** 10.5808/GI.2020.18.2.e22

**Published:** 2020-06-16

**Authors:** Ryota Yamada, Yuka Tatieisi

**Affiliations:** 1 Fuku Inc., Tokyo 113-0033, Japan; 2 National Bioscience Database Center, Japan Science and Technology Agency, Tokyo 102-8666, Japan

**Keywords:** dictionaries, Medical Subject Headings, natural language processing

## Abstract

The Medical Subject Headings (MeSH) thesaurus is a controlled vocabulary for indexing biomedical documents that is used for document retrieval and other natural language processing purposes. However, although the original English MeSH is freely available, its Japanese translation has a restricted license. We attempted to create an open alternative, and for this purpose we made a script for assigning MeSH UIDs to Japanese medical terms using Japanese-English glossaries. From the MeSpEn glossary and MEDUTX dictionary, we generated a 12,457-word Japanese-MeSH dictionary.

**Availability:** The script is available from" before the URL. https://github.com/roy29fuku/open-japanese-mesh.

## Introduction

The Medical Subject Headings (MeSH) [1] system is a controlled vocabulary developed and maintained by the United National Library of Medicine (NLM) that is used for indexing biomedical articles in PubMed.

MeSH is primarily used for indexing and searching the PubMed database, but it can also be used as a reliable dictionary of technical terms in the biomedical domain, as its headings and entry terms are representations of biomedical concepts approved by the NLM. Thus, MeSH is a valuable resource for natural language processing (NLP) applications. The metathesaurus in the Unified Medical Language Systems (UMLS) [2] includes translations of MeSH to several languages including Japanese.

However, although the original MeSH in English can be freely downloaded and used, the translations of MeSH in the UMLS are provided with “category 3” restrictions, which means that they cannot be incorporated into applications available outside the institution of the licensee. According to a mini-survey conducted in the 5th Biomedical Linked Annotation Hackathon (BLAH5) [3], although there are web-based dictionaries/thesauri that are freely consulted for finding MeSH UIDs or tree numbers by human readers, no dictionaries that are completely free for NLP applications are available.

## Methods

MeSH consists of three types of records: descriptors (main headings), qualifiers, and supplementary concept records. Descriptors are terms that characterize the subject matter. They are organized in a hierarchical structure based on broader/narrower relations of concepts. Qualifiers are used with descriptors and describe an aspect of a subject denoted by the descriptor. Supplementary concept records are names of chemicals, drugs, and new concepts. Supplementary concept records are not hierarchically ordered. Instead, each supplementary concept is linked to one or more descriptors. Descriptors and supplementary concepts have a heading (representative term) and entry terms (synonyms). Each record in MeSH is accompanied by an identifier (UID).

In order to link Japanese medical terms with medical concepts in MeSH, we used two open Japanese-English bilingual glossaries. The MeSpEn English-Japanese glossary is part of the MeSpEn multilingual medical glossary developed by the Text Mining Unit (TEMU) of the Barcelona Computing Center and available under the Creative Commons Attribution 4.0 International License [4]. The MEDUTX dictionary was developed by Kitasato University and is available from the Asia-Pacific Association for Machine Translation (AAMT) under a Creative Commons Attribution 3.0 International License [5]. The MeSpEn English-Japanese glossary has 16,756 unique Japanese terms and 10,738 unique English terms (27,668 unique pairs). The MEDUTX dictionary has 21,821 unique Japanese terms and 22,276 unique English terms (27,122 unique pairs). Merging the two dictionaries yielded a resource with 35,903 unique Japanese terms and 30,853 unique English terms (54,790 unique pairs).

We used the 2020 MeSH ASCII files for descriptors (d2020.bin) and supplementary concepts (c2020.bin) downloaded from the FTP site of the NLM on February 5, 2020. The descriptors file contained 242,205 terms (headings and entry terms) that were mapped to 29,640 concepts (UIDs) and the supplementary concepts file had 649,322 terms that were mapped onto 268,825 UIDs.

Since the Japanese-English dictionaries we used were much smaller than the MeSH vocabulary, we developed a Python script that can be applied to any Japanese-English glossary (in the form of tab-separated list of Japanese terms and corresponding English terms) and assigned the UIDs to Japanese terms where applicable, in order to be able to expand the output dictionary when more Japanese-English resources are available.

**Fig. 1.**
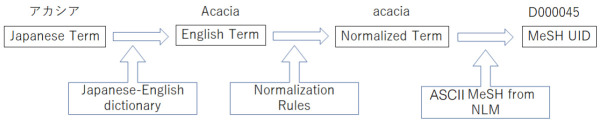
The UID assignment process.

We mapped Japanese medical terms to UIDs in the process illustrated in Fig. 1. First, a Japanese term was mapped to English term(s) with Japanese-English dictionary. The English terms were normalized as follows: they were placed in lowercase, *zenkaku* (full-width, non-ASCII) characters were converted to their *hankaku* (half-width, ASCII) counterparts, Greek characters were spelled out, and Roman numerals were converted into Arabic numerals. The jaconv library [6] was used for *zenkaku-to-hankaku* normalization. The MeSH terms were also normalized, and the normalized English terms from the dictionary were matched against the normalized MeSH terms.

The Python class for Japanese-English dictionaries, MeSH data, and normalization rules were defined in order to easily incorporate new dictionaries and new normalization rules. We also investigated the effect of each type of normalization.

## Results and Discussion

Without normalization of English terms, 2,838 out of 35,903 Japanese terms were mapped onto MeSH concepts (UIDs). With normalization of English terms, 12,457 Japanese terms out of 35,903 (about 34.7%) were mapped to UIDs. The contributions of each type of normalization are summarized in Table 1. The results show that case matching of the alphabet was the most effective normalization step, and the contributions of other types of normalization were small.

At least one Japanese term was assigned to 7,346 out of 298,465 MeSH concepts (UIDs), of which 6,185 were descriptors and 1,161 were supplementary concepts. This means that Japanese terms were assigned to about 20.9% (6,185/29,640) of descriptors and 0.4% (1161/268,825) of supplementary concepts.

Considering the size of the Japanese-English dictionary (about 3% of the MeSH vocabulary) this result seems reasonable. For improving its coverage, a list of translations of names of chemicals, drugs, and other named entities regarded as supplementary concepts in MeSH should be obtained.

## Conclusion

We made a script for assigning MeSH UIDs to Japanese medical terms using Japanese-English glossaries. From the MeSpEn glossary and MEDUTX dictionary, we obtained a 12,457-word Japanese-MeSH dictionary. This dictionary could be enhanced by using additional Japanese-English dictionaries. The script is available from https://github.com/roy29fuku/open-japanese-mesh under a Creative Commons Attribution 4.0 International License. Our future work includes a comparison with the Japanese translations in the UMLS metathesaurus.

## Figures and Tables

**Table 1.** Number of terms successfully assigned MeSH UIDs according to normalization

Normalization	Example	Mapped Japanese terms
None		2,838
Lowercasing	A → a	12,406
*Zenkaku*-to-*hankaku*	Ａ(\uFF21) →A (\u0041)	2,839
Greek-to-English	α→ alpha	2,857
Roman numerals-to-Arabic	VIII → 8	2,838
All		12,457
